# Self-perception of vocal fatigue and insomnia severity in teachers

**DOI:** 10.1590/2317-1782/e20240067en

**Published:** 2025-02-07

**Authors:** Jaírle Laís Alves do Nascimento, Felipe Silva de Araujo, Vanessa Veis Ribeiro, Juliana Fernandes Godoy, Larissa Thaís Donalonso Siqueira

**Affiliations:** 1 Departamento de Fonoaudiologia, Universidade Federal do Rio Grande do Norte – UFRN - Natal (RN), Brasil.; 2 Departamento de Fonoaudiologia, Universidade de Brasília – UNB - Brasília (DF), Brasil.

**Keywords:** Fatigue, Insomnia, Teachers, Self Assessment, Speech, Language and Hearing Sciences, Voice

## Abstract

**Purpose:**

To compare the self-perception of vocal fatigue and insomnia severity between teachers at risk and not at risk for dysphonia and between men and women.

**Method:**

The study included 120 female and 80 male teachers from various teaching levels. All participants completed self-assessment questionnaires on their working conditions, the Screening Index for Voice Disorder (SIVD), the Vocal Fatigue Index (VFI), and the Insomnia Severity Index (ISI). Teachers were grouped into those at risk (DG) and not at risk for dysphonia (NDG).

**Results:**

Both DG and NDG reported noise, stress, and dust in the work environment. These factors were more frequent in DG, which also scored above the cutoff for all VFI factors, while NDG scored high in Factor I and the total score but scored below the cutoff in Factor IV. Analysis per gender revealed no difference between DG and NDG among males, except for Factor IV. Among females, Factor IV scores were above the cutoff in DG. ISI indicated all participants had subthreshold insomnia.

**Conclusion:**

Teachers often had symptoms of vocal fatigue and subthreshold insomnia regardless of the risk for dysphonia. However, DG teachers had higher scores on both protocols. Also, DG females recovered from vocal fatigue symptoms after vocal rest, unlike their counterparts who were not at risk. Both DG and NDG males and females experienced vocal fatigue and limitations, but only those at risk recovered after vocal rest.

## INTRODUCTION

The human voice is an important communication tool, capable of expressing individual characteristics, including age group, personality, and sociocultural belonging^(1)^. The voice is also essential to some people’s occupations. Thus, dysphonia impacts quality of life and interferes directly with the ability to perform professional activities^(2)^.

Teachers are occupational voice users who frequently have vocal signs and symptoms. A 2018 study with basic education teachers revealed that 79.2% of the 634 participants reported some sign or symptom of voice changes, while only 20.8% stated being free from them^(3)^. The factors that help trigger vocal problems in this population include individual, environmental, and organizational characteristics, which cause or facilitate the development of dysphonia^(4,5)^, referred to as a Work-Related Voice Disorder (WRVD)^(2)^.

WRVDs are vocal deviations caused by professional activities, hindering or impairing the worker's performance and communication, with or without laryngeal lesions^(2)^. Due to their multifactorial nature, the development of WRVD involves occupational factors (directly linked to the work process), environmental factors (workplace characteristics), and individual factors (the person’s physical makeup, which may predispose them to develop vocal problems)^(2)^.

The teachers’ occupation demands intense vocal use, with many working multiple jobs and teaching for many hours a day in classrooms with inadequate infrastructure. These environments often include dust, smoke, humidity, poor ventilation, unfavorable acoustics, loud noise, and too many students^(5,6)^.

Unfavorable working conditions, such as those mentioned above, tend to lead teachers to speak at a higher intensity to compete with environmental noise^(6)^. However, prolonged loud voice use without proper training can cause excessive tension in the laryngeal muscles and increased compression force on the vocal folds, facilitating the occurrence of phonotrauma^(7)^ and contributing to the development of vocal problems. These conditions lead to higher rates of absenteeism, consequently resulting in significant financial costs and delays in school schedules^(6)^.

Dysphonia can lead to various complaints, with the most common among teachers being hoarseness, throat clearing, varied vocal emission, voice loss, bodily and/or respiratory discomfort, fatigue, and vocal exhaustion^(8)^. Vocal fatigue can be defined as a set of self-perceived vocal symptoms caused by increased phonatory effort associated with intense vocal demands or neuromuscular deficits^(9)^.

In addition to vocal fatigue symptoms, studies have shown that organizational and environmental workplace conditions help decline teachers’ sleep quality^(10,11)^. Sleep disorders are increasingly common, with insomnia being the most prevalent among the general population^(12)^. The causes are complex, involving factors such as mental health disorders, psychosocial aspects, inappropriate bedtime and bedroom habits and behaviors, and medical conditions^(13)^.

Considering the restorative role of sleep, changes in its quality can increase vocal fatigue symptoms, as a few hours of sleep may not be enough to restore the body's needs and maintain bodily balance^(14)^. This highlights the need for studies investigating the relationship between sleep and voice. Although teachers are the most studied professional group in the field of voice, there is a scarcity of research exploring the influences of teachers’ working conditions on vocal health, particularly regarding sleep, insomnia, and other related disorders^(15)^.

Considering the importance of adequate vocal health for teachers' professional performance and the negative impact of dysphonia on their quality of life^(16)^ and the students' cognitive learning performance^(17)^, it is essential to study the relationship between their working conditions and voice disorders and associate them with potential impacts on their quality of life and sleep.

Understanding the potential relationship between these elements will enable the development of public policies focused on teachers' health and well-being, thus generating positive effects on vocal health, quality of life, and education. This is crucial since teachers’ health issues directly impact the social and educational development of the country. Furthermore, the study’s findings may provide data that support and assist in creating laws to protect these workers’ health.

Hence, this study aimed to compare the self-perception of vocal fatigue and the severity of insomnia between teachers at risk and not at risk for dysphonia and between men and women.

## METHODS

This cross-sectional, analytical, quantitative study was submitted to the institution’s Research Ethics Committee and approved under evaluation report no. 5.498.404. All participants signed an informed consent form.

The study included teachers of both genders, over 18 years old, teaching any education level for at least six months. The study excluded teachers who had undergone laryngeal surgery, were currently undergoing otorhinolaryngological or speech-language-hearing treatment for voice or larynx, and had auditory complaints.

The teachers responded to the questionnaires online on the Google Forms^®^ digital platform from October 2022 to October 2023. The study was advertised on social networks and media to recruit teachers for participation.

The Screening Index for Voice Disorder (SIVD)^(18)^ assessed whether the teacher was at risk of developing dysphonia. This protocol addresses 12 vocal and laryngopharyngeal symptoms, whose frequency of symptoms teachers indicate as never, rarely, sometimes, or always. “Sometimes” and “always” score 1 point, and “never” and “rarely” score 0 points per symptom. The cutoff for identifying the risk of voice disorder is based on five symptoms. Thus, the sample was classified into two groups: Dysphonic Group (DG) – teachers of both genders with self-reported vocal complaints and within the risk range for dysphonia; and Non-Dysphonic Group (NDG) – vocally healthy teachers of both genders, with no self-reported complaints and outside the risk range for dysphonia.

The participating teachers responded to a questionnaire created by the authors regarding their working conditions (presence of noise, dust, smoke, humidity, stress, and rest areas at work; classroom acoustics, teaching pace, working hours, and teaching experience; vocal changes, seeking medical/speech-language-hearing care, and guidance on voice use) and sociodemographic data (age and gender).

The Vocal Fatigue Index (VFI) validated for Brazilian Portuguese^(19)^ analyzed the self-perception of vocal fatigue symptoms. The VFI is a self-perception instrument developed to identify individuals with this condition reliably^(19)^. It is divided into four factors: I – fatigue and vocal limitation; II – vocal restriction; III – physical discomfort associated with voice; IV – recovery with vocal rest; along with a total score. This provides a broad view of the impact of vocal fatigue and quantifies the associated symptoms. The frequency of the 17 vocal fatigue symptoms was analyzed using a Likert scale ranging from never (0) to always (4). The five factors were calculated according to the instructions from the authors who validated the VFI for Brazilian Portuguese, with cutoff values of 4.50 for factor I, 3.50 for factor II, 1.5 for factor III, 8.5 for factor IV, and 11.50 for the total score^(19)^.

The Insomnia Severity Index (ISI)^(20)^ investigated insomnia symptoms. The ISI was developed to measure the patient's perception of sleep difficulties (including subjective symptoms and the consequences of insomnia) and the degree of concern or distress caused by these difficulties^(20)^. Its score is calculated by simply summing the seven questions, with the result ranging from 0 to 28 points. Thus, insomnia can be classified as follows: 0 to 7 – no clinical evidence of insomnia; 8 to 14 – subthreshold insomnia, 15 to 21 – mild to moderate clinical insomnia; and 22 to 28 – severe insomnia^(20)^.

The data were analyzed descriptively and inferentially. Statistical analysis was performed using Jamovi 2.3.21.0 software. The data underwent normality testing using the Kolmogorov-Smirnov test, and the groups were compared with the Mann-Whitney test. The significance level was set at 5%. Descriptive statistics for the quantitative variables were presented in measures of variability (standard deviation) and central tendency (mean and median).

## RESULTS

The study sample included 200 teachers of both genders (120 women and 80 men), aged 21 to 66 years (mean age of 41.64 and standard deviation ±10.56 years), teaching from preschool to higher education. SIVD data indicated that 100 teachers were at risk, and 100 were not at risk for dysphonia. The sample characterization data are shown in Table 1.

**Table 1:** Characterization of the study sample of teachers (N = 200)

**Variables**	**Mean**	**SD**
Age – years	41.64	10.56
Time of teaching experience – years	15.93	13.51
Weekly workload	38.27	9.83
**Teaching level**	**Absolute frequency (n)**	**Relative frequency (%)**
Preschool/Kindergarten	25	12.5
Elementary/Middle school	58	29
High school	60	30
Higher education	57	28.5
**Gender**	**Absolute frequency (n)**	**Relative frequency (%)**
Males	80	40
Females	120	60

Caption: SD = standard deviation

**Figure 1:**
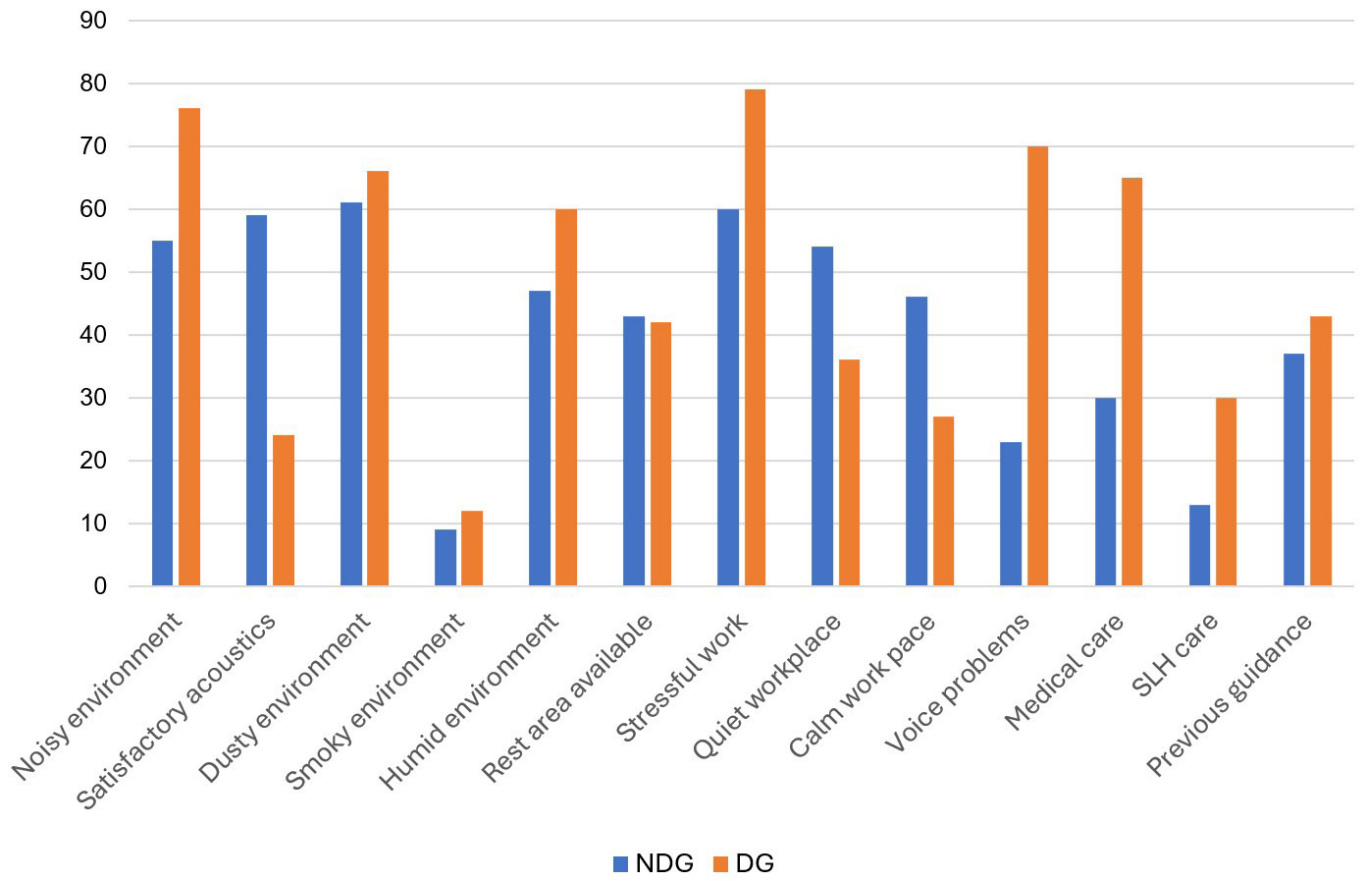
Main working conditions of teachers in the groups at risk (DG) and not at risk for dysphonia (NDG), in percentages

Figure 1 shows the percentage of the main self-reported working conditions of the teachers in the DG and NDG. Both groups reported high levels of noise, stress, and dust in the workplace, a hectic work pace, and no rest area – although the percentages were higher in the DG. The risk group also reported humidity, unsatisfactory classroom acoustics, and lack of tranquility in the workplace. Furthermore, 70% of the DG reported having voice problems and seeking medical care due to vocal issues.

Table 2 shows the vocal self-assessment values for DG and NDG. The median scores for all VFI and ISI domains differed significantly between the groups at risk and not at risk for dysphonia. The DG medians were above the cutoff (indicating self-perceived vocal fatigue) in all VFI factors, whereas the NDG medians were so only in factor I (vocal fatigue and limitation) and the total factor. Notably, the median for factor IV (recovery with vocal rest) was above the cutoff in the DG and below it in the NDG. The ISI indicated subthreshold insomnia in both groups but with greater severity in those at risk for dysphonia.

**Table 2:** Values ​​of the Screening Index for Voice Disorder, Vocal Fatigue Index, and Insomnia Severity Index for the groups at risk and not at risk for dysphonia

**Variable**	**Group**	**Q_1_**	**Median**	**Q_3_**	**Mean**	**SD**	**p-values**
**SIVD**	DG	6.00	8.00	9.00	7.82	1.92	< 0.001
	NDG	1.00	2.00	4.00	2.21	1.47	
**VFI - factor I**	DG	10.0	16.00	21.0	15.59	7.61	< 0.001
	NDG	2.00	6.00	11.0	6.57	4.76	
**VFI - factor II**	DG	3.00	5.00	8.00	5.48	3.58	< 0.001
	NDG	0.00	2.00	4.00	2.19	2.27	
**VFI - factor III**	DG	3.00	6.00	11.0	6.78	4.69	< 0.001
	NDG	0.00	1.00	3.25	2.03	2.70	
**VFI - factor IV**	DG	6.00	9.00	11.0	8.10	3.05	< 0.001
	NDG	1.75	6.00	9.00	5.38	4.16	
**VFI - total**	DG	22.8	31.00	40.0	31.75	13.04	< 0.001
	NDG	13.0	16.00	22.0	17.41	6.90	
**ISI**	DG	8.75	12.00	16.3	12.31	5.66	< 0.001
	NDG	5.75	9.50	12.0	9.52	5.03	

Mann-Whitney test p < 0.05

Caption: SIVD = Screening Index for Voice Disorder; VFI = Vocal Fatigue Index; ISI = Insomnia Severity Index; VFI factor I = Fatigue and vocal limitation; VFI - factor II = Vocal restriction; VFI - factor III = Physical discomfort associated with voice; VFI - factor IV = Recovery with vocal rest; DG = Group at risk for dysphonia; NDG = Group not at risk for dysphonia; Q_1_ = quartile 1; Q_3_ = quartile 3; SD = Standard deviation

Table 3 shows the self-assessment values for women and men in the DG and NDG. The median SIVD was above the cutoff for both men (37.5%) and women (58.3%) in the DG, which was not true for the NDG.

**Table 3:** Values ​​of the Screening Index for Voice Disorder, Vocal Fatigue Index, and Insomnia Severity Index for men and women in the groups at risk and not at risk for dysphonia

**Protocol**		**Women**				**Men**			
	**Group**	**Mean**	**Median**	**SD**	**p-value**	**Mean**	**Median**	**SD**	**p-value**
**SIVD**	DG	7.73	8.00	1.91	< 0.001	7.90	8.00	1.97	< 0.001
	NDG	2.42	2.50	1.53		2.00	2.00	1.39	
**VFI - factor I**	DG	15.57	16.00	8.25	< 0.001	14.83	14.00	6.16	< 0.001
	NDG	5.50	4.00	4.13		7.64	8.00	5.14	
**VFI - factor II**	DG	5.31	5.00	3.78	< 0.001	5.47	5.00	3.04	< 0.001
	NDG	1.54	0.00	1.95		2.84	3.00	2.40	
**VFI - factor III**	DG	7.19	8.00	4.74	< 0.001	5.53	4.50	4.38	< 0.001
	NDG	1.78	1.00	2.21		2.28	1.00	3.12	
**VFI - factor IV**	DG	8.13	9.00	3.08	< 0.001	7.87	8.00	2.92	0.691
	NDG	3.62	3.00	3.59		7.14	8.50	3.97	
**VFI - total**	DG	31.94	32.00	14.29	< 0.001	29.97	29.50	9.81	< 0.001
	NDG	17.20	16.00	5.42		17.62	18.00	8.17	
**ISI**	DG	12.19	11.00	5.84	0.048	12.17	13.50	5.04	0.016
	NDG	9.72	10.00	5.11		9.32	9.00	4.98	

Mann-Whitney test p < 0.05

Caption: SIVD = Screening Index for Voice Disorder; VFI = Vocal Fatigue Index; ISI = Insomnia Severity Index; VFI - factor I = Fatigue and vocal limitation; VFI - factor II = Vocal restriction; VFI - factor III = Physical discomfort associated with voice; VFI - factor IV = Recovery with vocal rest; DG = Group at risk for dysphonia; NDG = Group not at risk for dysphonia; SD = Standard deviation

Only VFI factor IV (recovery with vocal rest) was not statistically significantly different between DG and NDG males, although the NDG had a median above the cutoff. Moreover, NDG females had factor IV scores below the cutoff, whereas those of DG females were above it. Both men and women in both groups had total VFI and ISI scores above the cutoff, indicating subthreshold insomnia, with self-perception being more frequent and more severe in the DG.

## DISCUSSION

Teachers are the occupational voice users at greatest risk for dysphonia^(15)^. The causes of voice disorders in this population are multifactorial and include environmental, social, and emotional factors, ranging from their high vocal demand to the unfavorable physical characteristics and violence experiences in schools^(5,6)^, all of which negatively impact sleep quality^(11)^.

The results demonstrate that teachers experience vocal fatigue symptoms frequently, as the scores in both groups were higher than the cutoff for VFI factor I (vocal fatigue and limitation) and total factor. The DG scores were above the cutoff in all VFI factors. Having factor IV (recovery with vocal rest) scores above the cutoff indicates that teachers at risk for dysphonia can recover from vocal fatigue symptoms when they rest, which was not observed in the group not at risk. This result was unexpected and will be discussed below in this section.

A study applied the Vocal Signs and Symptoms Questionnaire (VSSQ) to university professors; those who reported more than two vocal signs and symptoms had a greater sense of fatigue^(21)^, showing that vocal symptoms can be indicators of vocal fatigue. This result may help explain the high VFI scores obtained by the group at risk for dysphonia in the present study. Vocal problems in teachers are related to factors ranging from environmental and organizational conditions to a lack of knowledge about proper voice use^(5,6)^. When examining the working conditions, both groups reported a hectic work pace, no rest area, and the presence of noise, dust, and stress, which may explain the vocal fatigue symptoms even in the group not at risk for dysphonia. Furthermore, the DG reported a lack of tranquility in the environment, unfavorable acoustics, and the presence of humidity, which may further impact vocal changes.

Excessive noise and poor acoustics in classrooms, in addition to the intense and constant voice use, contribute to vocal abuse, as teachers often have to speak louder to overcome the environmental noise^(6)^. Most of the sample in both groups reported not having received guidance on voice use, which contributes to their lack of preparation for handling the vocal load required by the job, promoting the development of vocal fatigue^(22)^. Furthermore, the lack of knowledge about vocal hygiene and economy makes it difficult for teachers to adopt strategies to protect their voice and avoid early wear on the laryngeal muscles – e.g., inadequate water intake and lack of vocal warm-ups and cool-downs before and after activities requiring prolonged vocal use^(5,6)^.

It is known that emotional stress can lead to musculoskeletal tension, which in turn can cause inappropriate behaviors during phonation, making it one of the main psychological factors associated with vocal symptoms^(23)^. This condition is common among teachers, and when combined with prolonged voice use, it can increase the self-perception of vocal fatigue^(24)^, which also results from mental and muscle fatigue^(25)^. Therefore, the high percentage of affirmative responses regarding stress at work by the DG and NDG may explain why both groups had vocal fatigue symptoms.

It is important to emphasize that, although both groups’ structural, environmental, and organizational working conditions were unfavorable for vocal health, the teachers at risk for dysphonia reported higher percentages. This likely contributed to a greater perception of vocal problems and fatigue in this group, as well as seeking medical care more often due to voice issues.

When analyzing the groups per gender, it was observed that the percentage of women at risk for dysphonia surpassed that of men who reported voice problems. Several factors influence the higher predisposition in females when it comes to the development of vocal issues, including biological, social, and psychological aspects^(4)^. Biological/physiological characteristics, such as lower levels of hyaluronic acid in the lamina propria of the vocal cords and differences in the glottic proportions of the larynx, contribute to a higher incidence of vocal problems in women^(26,27)^.

Similar to the total group (Table 2), all VFI factors were significantly different between DG and NDG for both male and female teachers, except for factor IV in the male group. These data align with other studies with teachers, which found that vocal overload, combined with organizational working conditions and the lack of proper vocal preparation, help increase vocal fatigue symptoms and a possible deficit in recovery^(21,22,28)^. This may explain the scores above the cutoff in factor I (fatigue and vocal limitation) and the total factor in the male group not at risk for dysphonia, despite being significantly different from the DG (Table 3). These findings highlight that vocal rest alone may not be sufficient to recover from fatigue symptoms, requiring strategies for prevention, health, and vocal training for this gender^(22,27,28)^.

Regarding factor IV (recovery with vocal rest), similar to the total group (Table 2), the female teachers at risk for dysphonia are able to recover from vocal fatigue symptoms when they take vocal rest – unlike the group not at risk, contributing to the total group results. One hypothesis is that the female teachers in the NDG have a lower perception of improvement with vocal rest (as they reported fewer vocal symptoms and less vocal fatigue) than those in the DG. On the other hand, a small amount of vocal rest may bring benefits and vocal comfort to these female teachers at risk for dysphonia, who have a higher perception of symptoms.

We also highlight that VFI factor IV in the male group was the only one that was not significantly different between teachers at risk and not at risk for dysphonia. A study with evangelical male pastors used the same self-perception protocols as the present study and found that improvement of vocal symptoms with rest was weakly correlated with dysphonia screening^(29)^. The authors also mentioned that one characteristic of the group was that most individuals were not at risk for dysphonia. Thus, we infer that the lack of difference in VFI factor IV between males in the two groups may be related to a lower perception of vocal complaints in men, which can make it harder to perceive improvement in the voice after rest. A similar observation was made in another study with teachers, which investigated vocal fatigue symptoms between a group that sought and one that did not seek speech-language-hearing therapy^(27)^. It found no statistical difference in factor IV between the groups, and the authors emphasized that one of the explanations could be the potential inconsistency in the responses of individuals without vocal changes, as it may be difficult to assess the recovery of unfelt issues^(27)^.

Regarding the self-perception of insomnia severity, all teachers’ scores, regardless of gender or the risk for dysphonia, were classified as subthreshold^(20)^. However, the DG had significantly higher scores than the NDG, which can also be justified by the teaching characteristics and conditions.

The cognitive origin of insomnia is related to the many stress factors that concern the individual, as daytime alertness causes bedtime hypervigilance^(13)^. The teachers’ occupational stress in this study sample was one of the factors with the highest percentage in both groups, with almost 80% of affirmative responses in the DG. In another study linking stress and sleep quality in university and healthcare professors, more than half of them reported poor sleep quality, with the factors most likely to cause stress being their long working hours and multiple tasks^(10)^.

Few studies have linked sleep and voice and/or the effect of these aspects on occupational voice users, such as teachers. A study with 862 individuals who completed three self-assessment instruments – the Epworth Sleepiness Scale (ESS), the Voice Handicap Index (VHI-10), and the Pittsburgh Sleep Quality Index (PSQI) – found that the worse the self-assessed vocal quality, the higher the scores in all the instruments, highlighting that the poorer the perception of sleep quality, the greater the perceived voice handicap^(30)^. The same study emphasizes that vocal fatigue can be caused by bodily fatigue due to sleep disorder or deprivation^(30)^. This may explain our study data, as all participants had sleep disorders and simultaneously reported vocal fatigue symptoms, with both being more evident in the DG.

The human voice has multidimensional characteristics, and self-assessment is only one of the many aspects that make up the clinical evaluation of this phenomenon. Hence, one limitation of this study was the lack of other objective voice assessments, which could contribute to a better understanding of the findings and their relationship with insomnia. Therefore, it is suggested that future studies verify associations using other assessments and analyze these outcomes per teaching level, whose working characteristics differ and may impact the voice and insomnia differently. Another limitation to consider is that this study had a convenience sample, which may hinder the generalization of the data.

Moreover, statistical methods such as correlation between the groups could help understand the study outcomes better. On the other hand, we highlight the strengths of this study, such as its sample size and the investigation of self-perception of insomnia, which may help develop public health actions to support the vocal health of these occupational voice users.

## CONCLUSION

Regardless of the risk for dysphonia, teachers had frequent vocal fatigue symptoms and subthreshold insomnia. However, those at risk for dysphonia had higher scores on both self-assessment protocols. Teaching conditions, combined with a lack of information about vocal health and well-being, may contribute to the onset of vocal fatigue, vocal symptoms, and insomnia. Additionally, female teachers at risk for dysphonia recover from vocal fatigue symptoms after vocal rest, which did not occur in female teachers not at risk. As for male teachers, both those at risk and those not at risk for dysphonia had vocal fatigue and limitations, with only the teachers at risk recovering after vocal rest.
