# Supplementary material for: Self-perception of vocal fatigue and insomnia severity in teachers
Source: Codas. 2025 Feb 7;37(2):e20240067. doi: 10.1590/2317-1782/e20240067en (PMC11813181; doi:10.1590/2317-1782/e20240067en)
Supplement: Translation

# Translation: Autopercepção de fadiga vocal e da gravidade da insônia em professores

**DOI:** 10.1590/2317-1782/e20240067pt

**Authors:** Jaírle Laís Alves do Nascimento, Felipe Silva de Araujo, Vanessa Veis Ribeiro, Juliana Fernandes Godoy, Larissa Thaís Donalonso Siqueira

**Affiliations:** 1 Departamento de Fonoaudiologia, Universidade Federal do Rio Grande do Norte – UFRN - Natal (RN), Brasil.; 2 Departamento de Fonoaudiologia, Universidade de Brasília – UNB - Brasília (DF), Brasil.

**Keywords:** Fadiga, Insônia, Docentes, Autoavaliação, Fonoaudiologia, Voz

## Abstract

**Objetivo:**

Comparar autopercepção de fadiga vocal e gravidade da insônia de professores com e sem risco para disfonia, bem como entre homens e mulheres.

**Método:**

Participaram 120 professoras e 80 professores, de vários níveis de ensino. Todos responderam aos questionários de autoavaliação: condições de trabalho docente, Índice de Triagem de Distúrbios da Voz-ITDV, Índice de Fadiga Vocal-IFV e Índice de Gravidade da Insônia-IGI. Os professores foram distribuídos em grupo com risco (GD) e sem risco para disfonia (GND).

**Resultados:**

GD e GND relataram ruído, estresse e poeira no ambiente de trabalho, sendo mais frequentes no GD. No IFV, GD apresentou escores acima dos valores de corte para todos os fatores, enquanto GND apresentou valores elevados para o fator-I e total, e valores abaixo da nota de corte para o fator-IV. Na análise por gênero, não houve diferença no grupo masculino entre GD e GND apenas para o fator-IV. Para as professoras, o fator-IV apresentou escore acima do valor de corte para GD. Quanto ao IGI, todos apresentaram insônia subliminar.

**Conclusão:**

Independente do risco para disfonia, professores apresentaram frequente sintomatologia de fadiga vocal e insônia subliminar, porém, professores com risco para disfonia apresentaram escores mais elevados para ambos os protocolos. Além disso, professoras com risco para disfonia recuperam sintomas de fadiga vocal após repouso vocal, o que não ocorreu com as professoras sem risco. Os professores com e sem risco para disfonia apresentaram fadiga e limitação vocal, sendo que apenas os professores com risco se recuperam após repouso vocal.

## INTRODUÇÃO

A voz humana é uma importante ferramenta de comunicação, capaz de expressar características próprias dos indivíduos que vão desde a faixa etária, à personalidade e ao pertencimento a um grupo sociocultural^(1)^. Para algumas pessoas, a voz representa o seu principal instrumento de trabalho. Dessa forma, além de impactos na qualidade de vida, a presença de uma disfonia causa interferências diretas na capacidade de desenvolver as atividades profissionais^(2)^.

Professores são profissionais da voz que apresentam com frequência sinais e sintomas vocais. Um estudo realizado em 2018 com docentes da educação básica demonstrou que dos 634 participantes, 79,2% relataram possuir algum sinal ou sintoma, enquanto apenas 20,8% afirmaram não ter qualquer alteração^(3)^. Os fatores que levam ao surgimento de problemas vocais nesta população englobam características individuais, ambientais e organizacionais que provocam ou mesmo favorecem o desenvolvimento da disfonia^(4,5)^, denominado de Distúrbio de Voz Relacionado ao Trabalho - DVRT^(2)^.

Os DVRT são desvios vocais que surgem como consequências da atividade profissional, que impedem ou comprometem a atuação e a comunicação do trabalhador, podendo ou não haver presença de lesões laríngeas^(2)^. Devido a sua característica multicausal, o desenvolvimento do DVRT compreende fatores ocupacionais, diretamente associados ao processo de trabalho; ambientais, ou seja, características presentes no ambiente onde o trabalhador desenvolve suas atividades laborais e individuais, relacionadas a constituição física do indivíduo que podem favorecer o surgimento do problema vocal^(2)^.

No caso dos professores, a profissão docente exige uma intensa demanda vocal, sendo que boa parte atua em mais de um cargo, lecionando muitas horas por dia em salas de aula com estrutura inadequada, com presença de poeira, fumaça, umidade, além de ventilação inapropriada, acústica desfavorável, alto nível de ruído e um número muito grande de alunos^(5,6)^.

Condições de trabalho desfavoráveis como as citadas anteriormente geram a tendência de que o professor fale em intensidade mais elevada para competir com o ruído ambiental^(6)^. No entanto, o uso da voz em *loudness* forte por um longo período sem o devido treinamento pode provocar tensão excessiva na musculatura da laringe e o aumento da força de compressão das pregas vocais, favorecendo a ocorrência de fonotraumas^(7)^ e ocasionando o desenvolvimento de problemas vocais. Essas condições favorecem o aumento do absenteísmo e, consequentemente, geram altos custos financeiros e atraso no cronograma das escolas^(6)^.

A presença de uma disfonia pode gerar diversas queixas, sendo as mais comuns em professores a rouquidão, pigarro, variações na emissão vocal, perda da voz, desconfortos corporais e/ou respiratórios, cansaço e fadiga vocal^(8)^. A fadiga vocal pode ser definida como um conjunto de sintomas vocais autopercebidos pelo indivíduo, que se manifesta em consequência do aumento do esforço fonatório associado a uma intensa demanda vocal ou déficit neuromuscular^(9)^.

Além de sintomas de fadiga vocal, estudos têm demonstrado que as condições organizacionais e ambientais do trabalho têm contribuído para a piora da qualidade do sono dos professores^(10,11)^. Transtornos do sono são cada vez mais comuns e dentre eles a insônia é o mais prevalente na população geral^(12)^. As causas são complexas e os fatores associados abarcam transtornos mentais e aspectos psicossociais, hábitos e comportamentos inadequados relacionados ao momento de dormir e ao ambiente do sono, além de condições médicas^(13)^.

Considerando o papel restaurador exercido pelo sono, alterações em sua qualidade podem influenciar o aumento de sintomas de fadiga vocal, visto que poucas horas de sono podem não ser suficientes para restaurar as necessidades do corpo e o equilíbrio corporal^(14)^. Esse fato expressa a necessidade de se realizar estudos que investiguem a relação entre sono e voz. Apesar dos professores serem a categoria profissional mais estudada na área da voz, observa-se uma escassez de estudos que buscam investigar as influências das condições do trabalho docente na saúde vocal e sobretudo no sono, no que diz respeito a insônia e demais transtornos^(15)^.

Levando em conta a importância de uma adequada saúde vocal para o desempenho profissional do professor e que a presença da disfonia produz repercussão tanto na qualidade de vida do profissional^(16)^, como afeta negativamente o desempenho cognitivo dos alunos no processo de aprendizagem^(17)^, é necessário que sejam realizados estudos para investigar a relação entre as condições de trabalho e a presença de distúrbios na voz e associar com os possíveis impactos na qualidade de vida e do sono desta população.

Compreender a eventual relação entre esses elementos possibilitará o desenvolvimento de políticas públicas voltadas à saúde e ao bem-estar do professor e assim gerar efeitos positivos na saúde vocal, na qualidade de vida e, consequentemente, na educação, uma vez que os agravos à saúde desse profissional têm impactos diretos no desenvolvimento social e educacional do país. Além disso, os resultados do estudo podem fornecer dados que respaldem e auxiliem a criação de leis que protejam a saúde destes trabalhadores.

Perante o exposto, o objetivo deste estudo foi comparar autopercepção de fadiga vocal e gravidade de insônia de professores com e sem risco para disfonia, bem como entre homens e mulheres.

## MÉTODO

Trata-se de um estudo transversal, analítico, de caráter quantitativo. O estudo foi submetido ao Comitê de Ética em Pesquisa da instituição e aprovado sob o parecer 5.498.404. Todos os participantes assinaram o Termo de Consentimento Livre e Esclarecido.

Participaram do estudo professores de ambos os gêneros, de qualquer nível de ensino, que estavam na profissão há mais de seis meses e com idade acima de 18 anos. Foram excluídos os professores que relataram ter realizado cirurgias na região de laringe, tratamentos otorrinolaringológicos ou fonoaudiológicos atuais para voz ou laringe e queixas auditivas.

Os professores responderam aos questionários em formato online, por meio da plataforma digital *Google Forms®* no período de outubro de 2022 a outubro de 2023. A divulgação da pesquisa foi realizada nas redes e mídias sociais a fim de recrutar os professores para participação do estudo.

Para verificar se o professor apresentava risco para desenvolver disfonia foi aplicado o protocolo Índice de Triagem de Distúrbios da Voz – ITDV^(18)^. Esse protocolo é composto por 12 sintomas vocais e laringofaríngeos. Os professores assinalaram a frequência dos sintomas em: nunca, raramente, às vezes e sempre. Atribui-se 1 ponto para as respostas “às vezes” ou “sempre” e 0 pontos para “nunca” ou “raramente”, para cada um dos sintomas. O ponto de corte para identificar o risco de distúrbio de voz é composto por cinco sintomas. Dessa forma, a amostra foi classificada em dois grupos: Grupo Disfônico (GD) - professores de ambos os gêneros com queixa vocal autorreferida e dentro da faixa de risco para a disfonia; e Grupo Não-Disfônico (GND) - professores de ambos os gêneros vocalmente saudáveis - sem queixa autorreferida e fora da faixa de risco para a disfonia.

Os professores participantes responderam a um questionário elaborado pelos autores sobre as condições de trabalho docente (presença de ruído, poeira, fumaça, umidade, estresse e local de descanso no trabalho; acústica da sala de aula, ritmo, carga horária de trabalho e tempo de docência; presença de alteração vocal, busca por atendimento médico/fonoaudiológico e orientação em relação ao uso da voz) e dados sociodemográficos (idade e gênero).

Para analisar a autopercepção dos sintomas de fadiga vocal foi utilizado o Índice de Fadiga Vocal – IFV, validado para o português brasileiro^(19)^. O IFV é um instrumento de autopercepção que foi desenvolvido com o objetivo de identificar de forma confiável os indivíduos com essa condição^(19)^. O protocolo é dividido em quatro fatores: I - fadiga e limitação vocal; II - restrição vocal; III - desconforto físico associado à voz; IV - recuperação com repouso vocal; além de um escore total. Dessa forma, o instrumento permite uma visualização ampla do impacto da fadiga vocal, além da quantificação dos sintomas associados. A frequência dos 17 sintomas de fadiga vocal foi analisada em uma escala de Likert que varia de nunca (zero) a sempre (quatro). O cálculo dos cinco fatores foi realizado conforme as instruções dos autores que validaram o IFV para o português brasileiro, sendo 4,50 o valor de corte para o fator I, 3,50 para o fator II, 1,5 para o fator III, 8,5 para o fator IV e 11,50 para o escore total^(19)^.

Para a investigação dos sintomas de insônia, foi utilizado o Índice de Gravidade da Insônia^(20)^. O IGI foi desenvolvido com o objetivo de mensurar a percepção do paciente em relação às dificuldades no sono, incluindo sintomas subjetivos e as consequências da insônia, bem como os grau de preocupação ou sofrimento causado por essas dificuldades^(20)^. A pontuação deste protocolo é realizada por somatória simples das sete questões, sendo que o resultado pode variar de 0 a 28 pontos. Dessa forma, a insônia pode ser classificada da seguinte maneira: de 0 a 7 sem evidências clínicas de insônia, 8 a 14 insônia subliminar, 15 a 21 insônia clínica leve a moderada, 22 a 28 insônia grave^(20)^.

Os dados foram analisados de forma descritiva e inferencial. A análise estatística foi realizada por meio do software Jamovi 2.3.21.0. Os dados passaram pela análise de normalidade por meio do teste Kolmogorov–Smirnov e comparação entre os grupos foi realizada por meio do teste de Mann-Whitney. Adotou-se um nível de significância de 5%. A descrição das variáveis quantitativas foi realizada por meio de medidas de variabilidade (desvio-padrão) e tendência central (média e mediana).

## RESULTADOS

Participaram do estudo 200 professores, do ensino infantil ao superior, de ambos os gêneros, sendo 120 mulheres e 80 homens, com idades entre 21 e 66 anos (média de 41,64 anos de idade e desvio padrão ±10,56). Em relação ao grupo total, 100 professores apresentaram risco para desenvolver disfonia e 100 não apresentaram risco a partir dos dados obtidos pelo protocolo ITDV. Os dados de caracterização da amostra encontram-se na tabela 1.

**Tabela 1:** Caracterização da amostra de professores estudados (N=200)

**Variáveis**	**Média**	**Dp**
Idade – anos	41,64	10,56
Tempo de atuação docente – anos	15,93	13,51
Carga horária semanal	38,27	9,83
**Nível de ensino**	**Frequência absoluta (n)**	**Frequência relativa (%)**
Ensino infantil	25	12,5
Ensino fundamental	58	29
Ensino médio	60	30
Ensino superior	57	28,5
**Gênero**	**Frequência absoluta (n)**	**Frequência relativa (%)**
Masculino	80	40
Feminino	120	60

Legenda: Dp = desvio padrão

A Figura 1 mostra, em porcentagem, as principais condições de trabalho autorrelatadas pelos professores do GD e do GND. Ambos os grupos relataram alto nível de ruído, presença de estresse e poeira no ambiente de trabalho, falta de tranquilidade no ritmo de trabalho e falta de lugar para descanso. Contudo, observa-se porcentagem consideravelmente mais elevada para o grupo com risco para disfonia. Além disso, o GD relatou presença de umidade, acústica da sala de aula não satisfatória e falta de tranquilidade no ambiente de trabalho. Ainda, em relação ao GD, 70% da amostra relatou apresentar problemas na voz e já ter buscado atendimento médico devido a problemas vocais.

**Figura 1:** Principais condições de trabalho docente dos grupos com risco para disfonia (GD) e sem risco para disfonia (GND), em porcentagem

A Tabela 2 mostra os valores obtidos nos protocolos de autoavaliação vocal de ambos os grupos, grupo com risco para disfonia (GD) e sem risco para disfonia (GND). Observa-se que a mediana dos escores de todos os domínios do protocolo IFV e do protocolo IGI apresentou diferença significativa na comparação dos grupos com e sem risco de disfonia. Para o GD, as medianas de todos os fatores do IFV foram acima do ponto de corte. Para o GND, somente para o fator I (fadiga e limitação vocal) do IFV e fator total a mediana foi acima do ponto de corte indicando autopercepção de fadiga vocal. Destaca-se que a mediana do fator IV (recuperação com repouso vocal) está acima do ponto de corte no GD e abaixo, no GND. O protocolo IGI indica insônia subliminar para ambos os grupos, porém com maior gravidade para aqueles com risco de disfonia.

**Tabela 2:** Valores dos protocolos Índice de Triagem dos Distúrbios da Voz, Índice de Fadiga Vocal e Índice de Gravidade da Insônia para o grupo com risco para disfonia (GD) e sem risco para disfonia (GND)

**Variável**	**Grupo**	**Q1**	**Mediana**	**Q3**	**Média**	**Dp**	**Valores de p**
**ITDV**	GD	6,00	8,00	9,00	7,82	1,92	< 0,001
	GND	1,00	2,00	4,00	2,21	1,47	
**IFV - fator I**	GD	10,0	16,00	21,0	15,59	7,61	< 0,001
	GND	2,00	6,00	11,0	6,57	4,76	
**IFV - fator II**	GD	3,00	5,00	8,00	5,48	3,58	< 0,001
	GND	0,00	2,00	4,00	2,19	2,27	
**IFV - fator III**	GD	3,00	6,00	11,0	6,78	4,69	< 0,001
	GND	0,00	1,00	3,25	2,03	2,70	
**IFV - fator IV**	GD	6,00	9,00	11,0	8,10	3,05	< 0,001
	GND	1,75	6,00	9,00	5,38	4,16	
**IFV - total**	GD	22,8	31,00	40,0	31,75	13,04	< 0,001
	GND	13,0	16,00	22,0	17,41	6,90	
**IGI**	GD	8,75	12,00	16,3	12,31	5,66	< 0,001
	GND	5,75	9,50	12,0	9,52	5,03	

Teste de Mann Whitney p<0,05

**Legenda:** ITDV = Índice de Triagem dos Distúrbios da Voz; IFV = Índice de fadiga vocal; IGI = Índice de Gravidade da Insônia; IFV - Fator I = fadiga e limitação vocal; IFV - Fator II = restrição vocal; IFV - Fator III = desconforto físico associado à voz; IFV - Fator IV = recuperação com repouso vocal; GD = Grupo com risco para disfonia; GND = Grupo sem risco para disfonia; Q1 = primeiro quartil; Q3 = terceiro quartil; Dp = desvio padrão

A Tabela 3 mostra os valores obtidos nos protocolos de autoavaliação das mulheres e homens em cada grupo estudado (GD e GND). No protocolo ITDV constatou-se mediana acima dos valores de corte para o GD, tanto para homens (37,5%) quanto para mulheres (58,3%), o que não foi observado no GND.

**Tabela 3:** Valores dos protocolos Índice de Triagem dos Distúrbios da Voz e Índice de Fadiga Vocal e Índice de Gravidade da Insônia para o grupo com risco para disfonia (GD) e sem risco para disfonia (GND) de mulheres e homens

**Protocolo**		**Mulheres**				**Homens**			
	**Grupo**	**Média**	**Mediana**	**Dp**	**Valores de p**	**Média**	**Mediana**	**Dp**	**Valores de p**
**ITDV**	GD	7,73	8,00	1,91	< 0,001	7,90	8,00	1,97	< 0,001
	GND	2,42	2,50	1,53		2,00	2,00	1,39	
**IFV - fator I**	GD	15,57	16,00	8,25	< 0,001	14,83	14,00	6,16	< 0,001
	GND	5,50	4,00	4,13		7,64	8,00	5,14	
**IFV - fator II**	GD	5,31	5,00	3,78	< 0,001	5,47	5,00	3,04	< 0,001
	GND	1,54	0,00	1,95		2,84	3,00	2,40	
**IFV - fator III**	GD	7,19	8,00	4,74	< 0,001	5,53	4,50	4,38	< 0,001
	GND	1,78	1,00	2,21		2,28	1,00	3,12	
**IFV - fator IV**	GD	8,13	9,00	3,08	< 0,001	7,87	8,00	2,92	0,691
	GND	3,62	3,00	3,59		7,14	8,50	3,97	
**IFV - total**	GD	31,94	32,00	14,29	< 0,001	29,97	29,50	9,81	< 0,001
	GND	17,20	16,00	5,42		17,62	18,00	8,17	
**IGI**	GD	12,19	11,00	5,84	0,048	12,17	13,50	5,04	0,016
	GND	9,72	10,00	5,11		9,32	9,00	4,98	

Teste de Mann Whitney p<0,05

Legenda: ITDV = Índice de Triagem dos Distúrbios da Voz; IFV = Índice de fadiga vocal; IFV - Fator I = fadiga e limitação vocal; IFV - Fator II = restrição vocal; IFV - Fator III = desconforto físico associado à voz; IFV - Fator IV = recuperação com repouso vocal; IGI = Índice de Gravidade da Insônia; GD = Grupo com risco para disfonia; GND = Grupo sem risco para disfonia; Dp = desvio padrão

Apenas o fator IV (recuperação com repouso vocal) do protocolo IFV, não apresentou diferença estatisticamente significativa entre os grupos masculinos (GD e GND), porém o GND apresentou mediana acima do valor de corte. Já para o grupo feminino, observou-se que o GND apresentou escore abaixo da nota de corte para o fator IV, enquanto as do GD apresentaram valores acima. Tanto os homens quanto as mulheres, de ambos os grupos, apresentaram escores acima do valor de corte para o fator total do IFV e para o protocolo IGI, o qual indica insônia subliminar, sendo que a autopercepção é mais frequente e com maior gravidade para o GD.

## DISCUSSÃO

Dentre os profissionais da voz, os professores são o grupo com maior risco para desenvolver disfonia^(15)^. A causa dos distúrbios de voz nessa população tem caráter multifatorial e incluem fatores ambientais, sociais e emocionais, que vão desde a alta demanda vocal a que eles são submetidos até as características desfavoráveis do ambiente físico de trabalho e às situações de violência vividas nas escolas^(5,6)^, impactando negativamente na qualidade de sono^(11)^.

De acordo com os resultados obtidos, é possível observar que professores possuem frequente sintomatologia de fadiga vocal, pois os escores de ambos os grupos foram maiores que os valores de corte para o fator I (fadiga e limitação vocal) e o fator total do protocolo IFV. Para o GD, todos os fatores do IFV apresentaram escores mais elevados que a nota de corte. Neste caso, o fator IV (recuperação com repouso vocal) estando acima do valor de corte indica que os professores com risco para disfonia conseguem recuperar os sintomas de fadiga vocal quando realizam repouso, o que não foi observado no grupo sem risco, resultado este que não era esperado e será discutido posteriormente nesta seção.

Em um estudo com professores universitários, em que foi aplicado o Questionário de Sinais e Sintomas Vocais (QSSV), os docentes que apresentaram mais de dois sinais e sintomas vocais tiveram maior sensação de fadiga^(21)^, demonstrando que a presença de sintomas vocais podem ser indicadores da fadiga vocal. Tal resultado pode justificar os elevados escores obtidos no IFV pelo grupo com risco para disfonia no presente estudo. A ocorrência de problemas vocais em professores está relacionada a fatores que vão desde as condições ambientais e organizacionais nos quais o docente é exposto até a falta de conhecimento em relação ao uso da voz^(5,6)^. Quando observadas as condições de trabalho, ambos os grupos relataram não possuir um ritmo de trabalho tranquilo, sem local de descanso, com presença de ruído, poeira e estresse, o que pode explicar a presença de sintomas de fadiga vocal também no grupo sem risco para disfonia. Ressalta-se ainda que o GD revelou falta de tranquilidade no ambiente, acústica desfavorável e presença de umidade, os quais podem impactar ainda mais na presença de alterações vocais.

Somado ao uso intenso e constante da voz, o excesso de ruído e a pobre acústica das salas de aula favorecem o abuso vocal, pois frequentemente o docente se vê na necessidade de falar mais forte para sobrepor a intensidade de sua voz ao ruído do ambiente^(6)^. Observa-se que a maior parte da amostra de ambos os grupos revelou não ter recebido orientação em relação ao uso da voz, fato que contribui para o despreparo no enfrentamento à carga vocal exigida pelo trabalho, favorecendo o desenvolvimento da fadiga vocal^(22)^. Além disso, a falta de conhecimento sobre higiene e economia vocal dificulta que os professores adotem estratégias para proteger a voz e assim evitar o desgaste precoce da musculatura da laringe, como a pouca ingestão de água e a não realização de aquecimento e desaquecimento vocal antes e depois de atividades que exijam uso vocal prolongado^(5,6)^.

Sabe-se que o estresse emocional pode ocasionar tensão musculoesquelética e assim provocar comportamentos inadequados durante a fonação, sendo um dos principais fatores psicológicos associados à ocorrência de sintomas vocais^(23)^. Tal condição é um problema frequente entre os professores e quando associado ao uso prolongado da voz pode aumentar a autopercepção de fadiga vocal^(24)^, decorrente também de uma fadiga mental e muscular^(25)^. Sendo assim, a alta porcentagem de respostas afirmativas em relação a presença de estresse no trabalho relatadas tanto pelo GD como pelo GND pode justificar ambos os grupos terem apresentado sintomatologia de fadiga vocal.

É importante salientar que, apesar dos dois os grupos terem apresentado condições estruturais, ambientais e organizacionais de trabalho desfavoráveis à saúde vocal, os professores com risco para disfonia apresentaram porcentagens mais elevadas, o que muito pode ter refletido em uma maior percepção de problema vocal e fadiga por este grupo, além da maior busca por atendimento médico devido a questões relacionadas à voz.

Quando realizada a análise dos grupos em relação ao gênero, foi possível observar que a porcentagem de mulheres com risco para disfonia se sobressai a de homens que relataram ter problemas na voz. Vários fatores influenciam na maior predisposição no gênero feminino no que se trata ao desenvolvimento de problemas vocais, dentre eles aspectos biológicos, sociais e psíquicos^(4)^. Biologicamente, características fisiológicas, como a menor presença de ácido hialurônico na lâmina própria das pregas vocais e as diferenças na proporção gótica da laringe favorecem a maior ocorrência de problemas vocais nas mulheres^(26,27)^.

Quanto ao protocolo IFV, assim como para o grupo total (Tabela 2), todos os fatores apresentaram diferença significativa entre GD e GND, tanto para professores quanto para professoras, exceto para o fator IV para o grupo masculino. Os dados obtidos concordam com outros estudos realizados com professores, nos quais foi observado que a sobrecarga vocal associada a condições organizacionais do trabalho e a falta de preparo vocal adequado contribui para aumento nos sintomas de fadiga vocal e um possível déficit na recuperação^(21,22,28)^. Tal fato pode ter refletido nos escores acima do valor de corte para o fator I (fadiga e limitação vocal) e fator total no grupo masculino sem risco para disfonia, mesmo apresentando diferença significativa quando comparado ao GD (Tabela 3). Esses dados evidenciam que apenas o repouso vocal pode não ser suficiente para a recuperação dos sintomas de fadiga, sendo necessário a adoção de estratégias de prevenção, saúde e treinamento vocal para este gênero^(22,27,28)^.

Em relação ao fator IV, o qual investiga a recuperação com repouso vocal, observamos que, assim como o grupo total (Tabela 2), as professoras com risco para disfonia conseguem recuperar os sintomas de fadiga vocal quando realizam repouso, o que não foi observado no grupo sem risco e contribuiu para os resultados obtidos no grupo total. Uma hipótese seria de que as professoras do GND têm menor percepção de melhora com repouso vocal, uma vez que apresentaram menor frequência de sintomas vocais e de fadiga vocal, diferente do GD. Por outro lado, um pouco de repouso vocal pode trazer benefícios e conforto fonatório para essas professoras com risco para disfonia, as quais apresentam maior percepção de sintomas.

Destacamos também que o fator IV do IFV no grupo masculino foi o único que não apresentou diferenças significativas quando comparados professores com e sem risco para disfonia. Em um estudo realizado com pastores evangélicos do gênero masculino, o qual utilizou os mesmos protocolos de autopercepção do presente estudo, observou fraca correlação entre a melhora dos sintomas vocais com o repouso e o rastreio da disfonia^(29)^. As autoras referiram ainda que uma das características do grupo era que a maioria dos indivíduos não apresentava risco para a disfonia. Dessa forma, inferimos que a não diferença entre os grupos no gênero masculino em relação ao fator IV do IFV, pode estar relacionada a menor percepção de queixas vocais no gênero masculino, o que pode dificultar a percepção de melhora na voz após o descanso. Algo similar foi verificado em um outro estudo com professores, que investigou a sintomatologia de fadiga vocal entre um grupo que procurou atendimento fonoaudiológico e outro que não buscou tratamento^(27)^. No referido trabalho, não foi observada diferença estatística para o fator IV entre os grupos e as autoras ressaltaram que uma das explicações é a possível incoerência nas respostas dos indivíduos sem alteração vocal, visto que pode ser difícil avaliar a recuperação de algo que não é sentido^(27)^.

Acerca da autopercepção da gravidade da insônia, observou-se que todos os professores, independentemente do risco para disfonia ou gênero, apresentaram escores que classificam a gravidade da insônia como subliminar^(20)^. No entanto, quando comparados os escores dos dois grupos, o GD apresentou valores bem mais elevados, o que também pode ser justificado pelas características e as condições de trabalho docente.

Cognitivamente, a origem da insônia está relacionada à grande quantidade de fatores estressantes que preocupam o indivíduo, em que o estado de excitação desenvolvido durante o dia resulta em uma hipervigilância que perdura até o momento de dormir^(13)^. Na amostra de professores que compõem o nosso estudo, a presença de estresse no trabalho foi um dos fatores que apresentou maior porcentagem em ambos os grupos, atingindo quase 80% de respostas afirmativas no GD. Em um outro estudo que relacionava o estresse e a qualidade do sono em professores do ensino superior e da área da saúde, mais da metade dos professores avaliados apresentaram qualidade de sono ruim, sendo que os fatores com maiores potenciais de estresse foram as longas jornadas de trabalho e a multiplicidade de tarefas exigidas ao educador^(10)^.

Destaca-se a reduzida quantidade de estudos que relacionam sono e voz e/ou o efeito desses aspectos nos grupos denominados profissionais da voz, como os professores. Um estudo, realizado com 862 indivíduos que responderam três instrumentos de autoavaliação – a Escala de Sonolência de Epworth (ESS), o Voice Handicap Index (VHI-10) e o Índice de Qualidade do Sono de Pittsburgh (PSQI) – observou que quanto pior a autoavaliação vocal, maiores eram os escores em todos os instrumentos, evidenciando que quanto pior a percepção de qualidade do sono maior a desvantagem vocal percebida^(30)^. O mesmo estudo ressalta que a fadiga vocal pode ser causada pela fadiga corporal devido a um distúrbio ou privação do sono^(30)^. O que pode justificar os dados obtidos no nosso estudo, visto que todos os participantes apresentaram alterações no sono e ao mesmo tempo sintomatologia de fadiga vocal, sendo ambos mais evidentes nos grupos disfônicos.

Por fim, considerando as características multidimensionais da voz humana e que a autoavaliação compõe apenas um dos vários aspectos que constituem a avaliação clínica desse fenômeno, uma das limitações deste estudo foi a não realização de outras avaliações objetivas da voz que poderiam contribuir para melhor compreensão dos achados e a relação com a insônia. Dessa forma, sugere-se que estudos futuros possam realizar as associações com outras avaliações e que esses desfechos sejam analisados por níveis de ensino, uma vez que as características do trabalho docente são diferentes e podem impactar na voz de formas distintas, bem como a insônia. Outra limitação que podemos considerar é que este estudo foi realizado com uma amostra por conveniência, o que pode dificultar a generalização dos dados.

No mais, métodos estatísticos como a correlação entre os grupos poderiam contribuir para melhor compreensão dos desfechos analisados nesse estudo. Em contrapartida, destacamos os pontos fortes do presente estudo como o tamanho da amostra e a investigação da autopercepção da insônia, a qual poderá contribuir para o desenvolvimento de ações públicas que respaldem a saúde vocal desses profissionais da voz.

## CONCLUSÃO

Independente do risco para disfonia, professores apresentaram frequente sintomatologia de fadiga vocal e insônia subliminar, porém, professores com risco para disfonia apresentaram escores mais elevados para ambos os protocolos de autoavaliação. Condições de trabalho docente somado à falta de informações quanto aos cuidados de saúde e bem-estar vocal podem contribuir para o aparecimento de fadiga vocal, sintomas vocais e a presença de insônia. Além disso, professoras com risco para disfonia recuperam sintomas de fadiga vocal após repouso vocal, o que não ocorreu com as professoras sem risco. Já os professores com e sem risco para disfonia apresentaram fadiga e limitação vocal, sendo que apenas os professores com risco se recuperam após repouso vocal.
